# Krüppel-like factor 15 in liver diseases: Insights into metabolic reprogramming

**DOI:** 10.3389/fphar.2023.1115226

**Published:** 2023-03-02

**Authors:** Hao Chen, Lan-Lan Li, Yan Du

**Affiliations:** ^1^ Department of Pharmacy, The First Affiliated Hospital of Anhui Medical University, Hefei, Anhui, China; ^2^ The Grade 3 Pharmaceutical Chemistry Laboratory of State Administration of Traditional Chinese Medicine, Hefei, Anhui, China

**Keywords:** liver diseases, metabolic reprogramming, Krüppel-like factor 15, gluconeogenesis, lipid, amino acid catabolism, bile acids, endobiotic metabolism and xenobiotic metabolism

## Abstract

Liver diseases, characterized by metabolic disorder, have become a global public health problem with high morbidity and mortality. Krüppel-like factor 15 (KLF15) is a zinc-finger transcription factor mainly enriched in liver. Increasing evidence suggests that hepatic KLF15 is activated rapidly during fasting, and contributes to the regulation of gluconeogenesis, lipid, amino acid catabolism, bile acids, endobiotic and xenobiotic metabolism. This review summarizes the latest advances of KLF15 in metabolic reprogramming, and explore the function of KLF15 in acute liver injury, hepatitis B virus, and autoimmune hepatitis. which aims to evaluate the potential of KLF15 as a therapeutic target and prognostic biomarker for liver diseases.

## Introduction

Krüppel-like factors (KLFs) were a subclass of zinc-finger DNA-binding transcription factors involved in a variety of biological functions such as homeostasis, metabolism, cellular proliferation, differentiation, inflammation, apoptosis and development (Bieker, 2001; McConnell and Yang, 2010; Presnell et al., 2015). In mammals, 18 members of the KLF family (KLF1–KLF18) have been identified. The most frequently encountered zinc-finger motif was the Cys2/His2 type linked by the TGEKP(Y/F) X amino acid sequence at their C-terminus (Chang et al., 2017; Oishi and Manabe, 2018). Among them, the Zn atom was tetrahedrally coordinated by two conserved cysteine and histidine residues, thus folding into a ββα structure (Dang et al., 2000). The regions outside the Zn-finger domain were unique (Brayer and Segal, 2008; McConnell and Yang, 2010). Nuclear localization and DNA binding were facilitated by Zn-finger motifs. The structural and functional differences of KLFs based on their highly variable N-terminal domains, which regulated protein interactions and transcription (Turner and Crossley, 1999). As a consequence, they can act as transcriptional activators, repressors, or both if they interact with high GC content DNA sequences, including 5′-CACCC-3′ motif at the promoters (Kaczynski et al., 2003). Additionally, KLFs regulated transcription by recruiting or sequestering various corepressors and activators, including cAMP response element binding protein (CREB), SIN3 transcription regulator family member A (Sin3A), p300, and others (McConnell and Yang, 2010; Kim et al., 2017; Fan et al., 2018). Therefore, KLFs dysfunction may be closely related to a variety of pathologies given their widespread nature and expression.

Krüppel-like factor 15 (KLF15) was a member of KLFs family and was abundantly expressed in the liver (Teshigawara et al., 2005; Takashima et al., 2010). Moreover, immunohistochemistry showed that hepatic KLF15 was expressed in skeletal muscle, stellate cells, and fibroblasts (Uchida et al., 2000; Uchida et al., 2001). It has been reported that hepatic KLF15 was an important transcriptional regulator of various physiological and pathological progresses, such as systemic glucose homeostasis, lipid flux and utilization, amino acid synthesis, etc. (Uchida et al., 2000; Haldar et al., 2007; Mallipattu et al., 2017; Han et al., 2019). A recent study reported alcohol disrupted whole-body metabolism for several days and increased KLF15 protein expression. Although mepiridone effectively inhibited alcohol-mediated elevation of serum corticosterone, KLF15 mRNA can still be induced by alcoholism, but to a lesser extent, suggesting a potential role of KLF15 in alcohol-related liver diseases (Tice et al., 2022). Another research showed that KLF15 level was elevated in liver maturation, whereas inhibition of KLF15 expression reduced liver maturation marker genes level. Similarly, KLF15 suppressed cell proliferation *via* inducing the level of cyclin inhibitor p57 ^cdkn1c^ in human induced pluripotent stem cells (iPSCs)-derived hepatoblasts (Anzai et al., 2021). In addition to these features, hepatic KLF15 also induced insulin secretion, tissue insulin sensitivity, and glucose uptake by regulating the insulin-sensitive glucose transporter type 4 (GLUT4) and peroxisome proliferator-activated receptor gamma (PPARγ) (Gray et al., 2002). Furthermore, KLF15 regulated endobiotic (steroids and bile acids) and xenobiotic (drugs and toxins) metabolism in the liver (Han et al., 2019). By upregulating branched-chain amino acid breakdown enzymes, KLF15 inhibited lipogenesis and promoted gluconeogenesis during fasted state (Fan et al., 2018). Interesting, KLF15 also plays a critical role in the circadian rhythmicity of amino acid metabolism and nitrogen detoxification in mammals (Teshigawara et al., 2005; Jeyaraj et al., 2012). Taken together, KLF15 was involved in metabolic diseases by regulating gluconeogenesis, lipid, amino acid catabolism, bile acids, endobiotic and xenobiotic metabolism.

Herein, we summarize the latest advances about roles of KLF15 in the hepatic metabolism disorder and explore how KLF15 coordinates complex physiologic responses in acute liver injury, hepatitis B virus, and autoimmune hepatitis, hoping to provide a theoretical basis for KLF15-targeted therapy for liver diseases.

### Structure and function of KLF15

**FIGURE 1:**
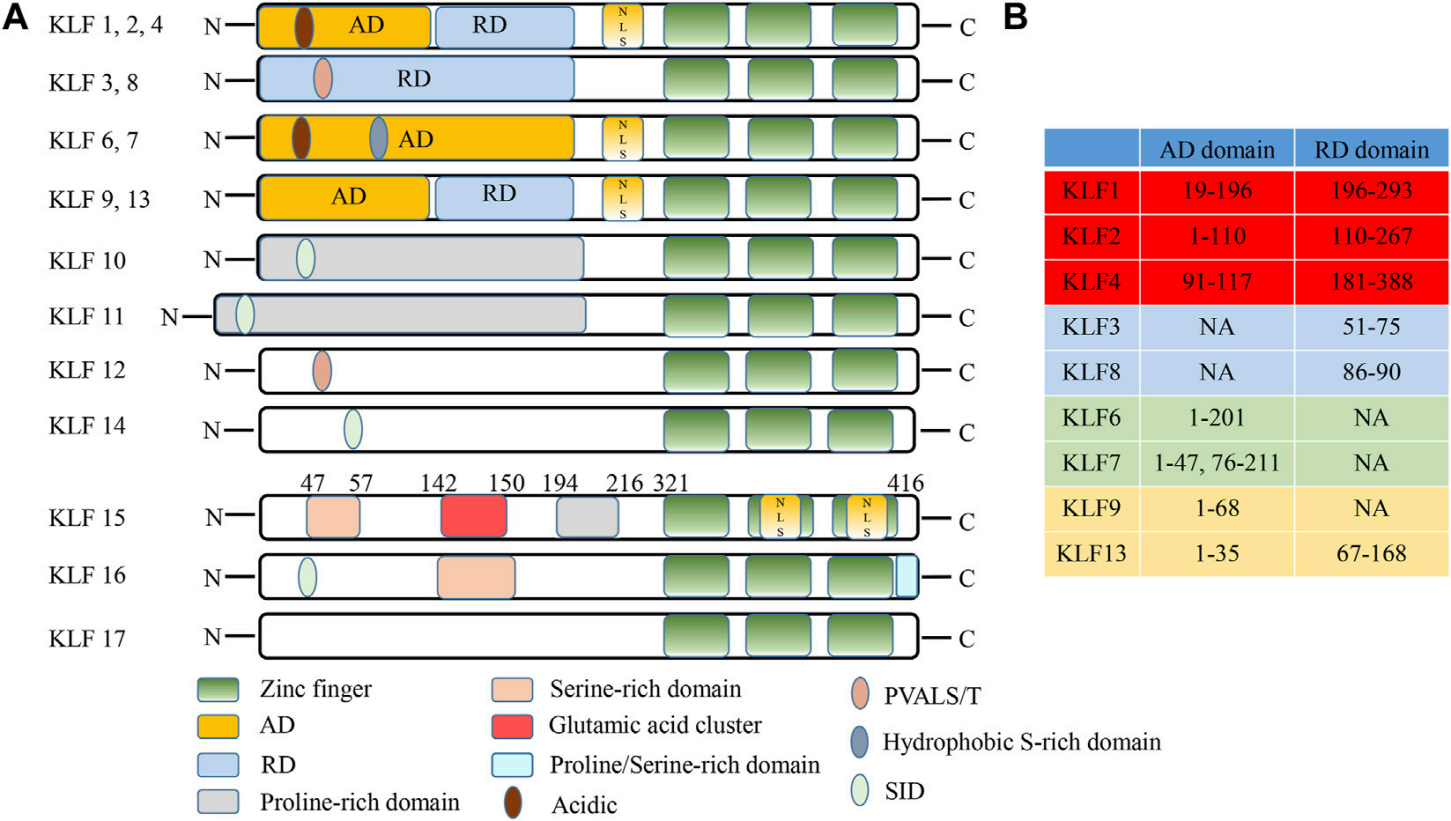
Schematic illustration of KLF family structures and domains (Adapted from Zhuoxiao Cao, Xinghui Sun, Basak Icli, et al. Role of Kruppel-like factors in leukocyte development, function, and disease. Blood, 2010, 116 (22):4404-14). **(A)** The structural characteristics of KLF family. **(B)** Differences in the AD/RD of partial KLF protein. AD, activation domain; RD, repression domain; NES, nuclear export signal; NLS, nuclear localization signal; N, N-terminus; C, C- terminus; PVALS/T, repression motif interacted with the corepressor CtBP2; SID, sin3 interaction domain; NA, not available.

Basing on human chromosome mapping, KLF15 was located at 3q21.3. KLF15 cDNA encoded a polypeptide of 416 amino acids and involved a single open reading frame of 1,248 bp (Adachi et al., 1994; Uchida et al., 2000; Uchida et al., 2001). Like other KLF family members, KLF15 protein comprised the conserved C_2_H_2_ zinc finger domains in the C-terminal region, that regulated transcription by binding to GC-rich sequences at target gene promoters (Uchida et al., 2000; Black et al., 2001; Kim et al., 2019). With the exception of the C_2_H_2_ zinc finger domains, the KLF15 coding sequence has little resemblance to other reported zinc finger genes (Kadonaga et al., 1987). For instance, there was no Krüppel, leucine zipper, Ets, or basic helix-loop-helix domain. Nevertheless, KLF15 comprised serine-rich or proline-rich sequences and many transcription factors used these motifs to transactivate genes and were therefore considered repression motifs, like those found in inhibitors like Krüppel and Wilms Tumor 1 (WT1) (Guo et al., 2018). Additionally, KLF15 has a glutamic acid cluster in amino acid residues 142 to 150. Consequently, glutamic acid clusters may be inhibitory elements in KLF15 (Figure 1) (Uchida et al., 2000). Although the KLFs family have similar DNA-binding capacity, they can be divided into three groups based on transcriptional activation or inhibition domains of the N-terminal region. The first group: acting as a transcriptional repressor with binding to C-terminal binding protein (CtBP); The second group: working as a transcriptional activator; The third group: serving as a transcriptional repressor *via* an α-helical motif (Rane et al., 2019). However, the interaction domain between KLF15 and protein has not been determined, and the classification of KLF15 remains to be further explored.

### Role of KLF15 in liver development

The liver was the largest organ in the body and plays a key role in maintaining homeostasis (Trefts et al., 2017; Ober and Lemaigre, 2018). Due to the liver has a high regenerative capacity, when the liver was injured with drug or alcohol, liver cells begin to proliferate, thereby restoring the size and function of the organ (Kung and Forbes, 2009; Chen et al., 2020; Campbell et al., 2021). During development, the hepatoblasts were produced from the posterior foregut endoderm, which only functions as a hematopoietic organ and has little metabolic function (Kung and Forbes, 2009). Around mouse E8.0, liver and pancreas domains were discriminated by the regulation of some cytokines. Subsequently, the liver bud was formed and then some signaling molecules (BMPs, FGFs, KLFs) drive hepatoblast proliferation and migration at E8.5-E9.5. Around E11.5-E13.5, that the hepatocytes became mature and then begin to produce a variety of metabolic enzymes and proteins required for adult liver function (Kamiya et al., 1999; Kamiya et al., 2002; Ober and Lemaigre, 2018; Campbell et al., 2021).

**FIGURE 2:**
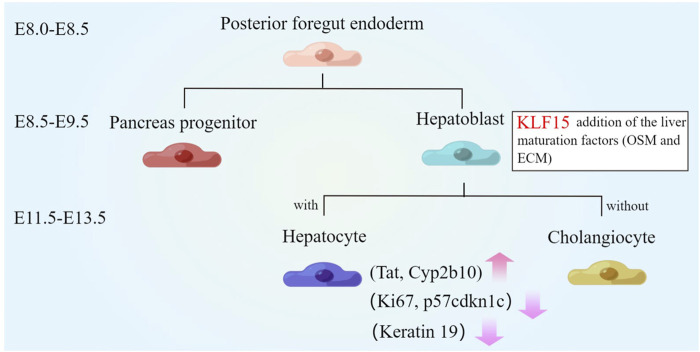
Role of KLF15 in liver development. The liver parenchymal progenitor cells, known as hepatoblasts, are specified from the posterior foregut endoderm from which pancreas progenitors are also derived. Around E11.5-E13.5, KLF15 expression is upregulated. The combination of KLF15 overexpression and liver maturation factors (OSM and extracellular matrices) significantly induce the expression of mature hepatocyte markers (Tat and Cyp2b10) and inhibit the expression of keratin 19. Furthermore, KLF15 inhibits the proliferation of human IPSC-derived hepatocytes, manifested by the decreased expression of Ki67 and the increased expression of p57^cdkn1c^.

Compared with other members of the KLFs family (KLF 5, 10, and 12), only KLF15 can promote liver maturation (Anzai et al., 2021). KLF15 was almost not expressed in fetal liver, but the expression of KLF15 was increased with liver development (Fan et al., 2018). Overexpression of KLF15 induced liver function gene levels at fetal mouse liver or human iPSCs-derived hepatocytes. Moreover, liver maturation factor oncostatin M (OSM) and extracellular matrix (ECM) were commonly used to induce hepatic maturation (Kamiya et al., 2002; Sekiya and Suzuki, 2011). Cultured with OSM and ECM for 7 days, KLF15 level was increased about 5 times compared with E13 primary hepatocytes, while other KLF family genes showed little changed. In addition, upregulation of KLF15 observably increased mature hepatocyte markers levels, including tyrosine aminotransferase (Tat) and cytochrome P450 2b10 (Cyp2b10) after co-culture with liver maturation factor. Furthermore, even without OSM and ECM, KLF15 can still partially induce liver function gene levels, such as suppressing the expression of keratin 19, thus promoting hepatocyte and inhibiting cholangiocytic differentiation (Anzai et al., 2021). On the other hand, cell proliferation was known to be downregulated during various cell differentiation processes (Zhu and Skoultchi, 2001; Brown et al., 2003). This may be related to the regulation of KLF15 (Ray and Pollard, 2012; Gao et al., 2017). KLF15 inhibited the proliferation of human IPSC-derived hepatocytes, manifested by the decreased level of Ki67 and the increased level of p57^cdkn1c^ (Anzai et al., 2021) (Figure 2). Therefore, KLF15 may be an important molecule in regulating the differentiation and development of liver.

### The role and mechanism of KLF15 in hepatic metabolism

Previous studies have confirmed that hepatic KLF15 was rapidly increased during fasting, and was involved in the regulation of hepatic gluconeogenesis, lipid, amino acid catabolism, bile acids, endobiotic and xenobiotic metabolism (Gray et al., 2007; Fan et al., 2018; Han et al., 2019; Wang et al., 2022).

### KLF15 and fasting

**FIGURE 3:**
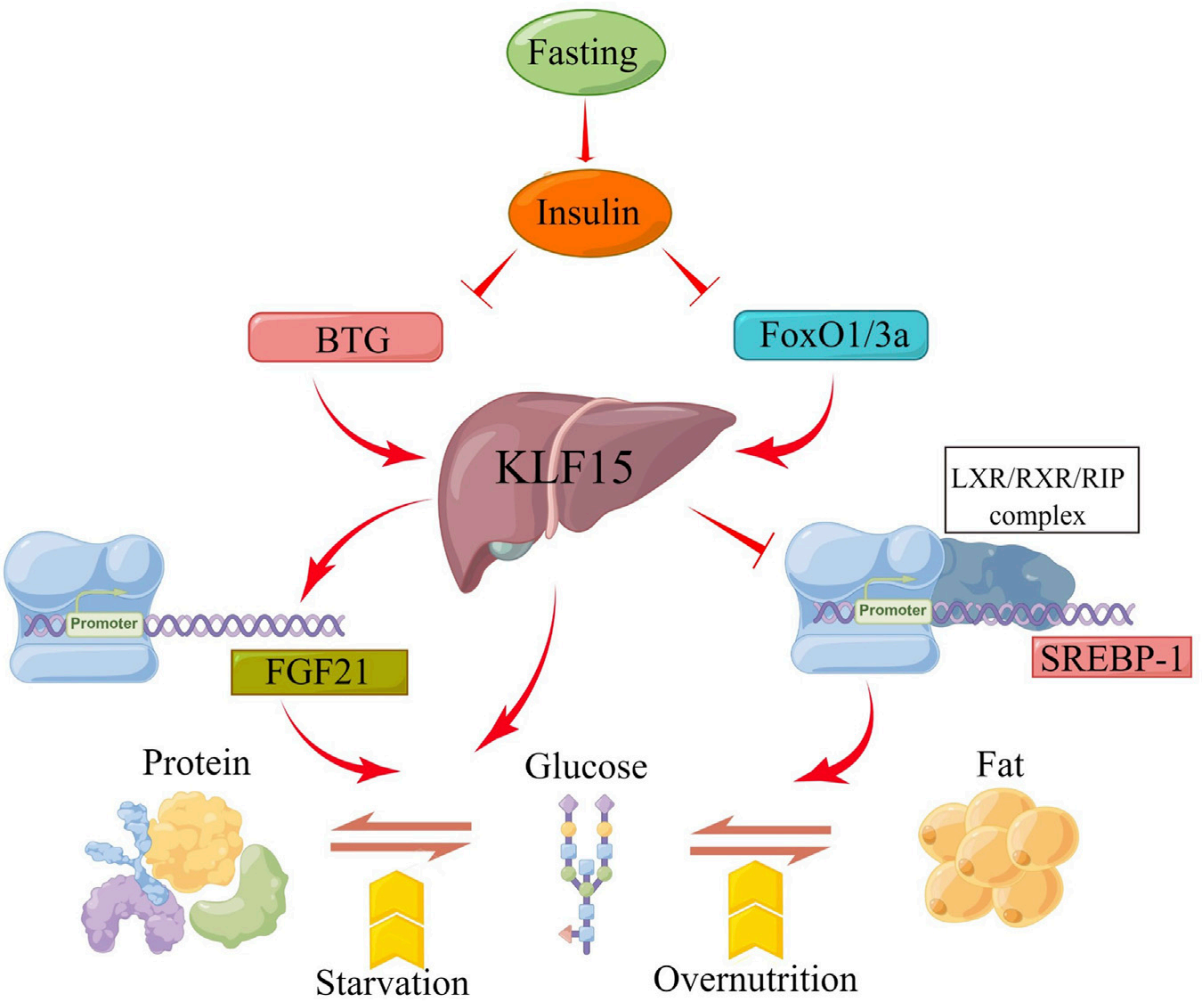
Schematic presentation of the molecular mechanism by which the KLF15 axis integrates metabolism. Fasting induces insulin secretion and then increases KLF15 expression, which is regulated by BTG and FOXO1/3A genes. Moreover, KLF15 is involved in the transformation of the three macronutrients (protein, carbohydrate, and fat) by targeting FGF21, PEPCK and SREBP-1 expression during fasting.

Fasting caused a number of complex adaptive metabolic responses (Maughan et al., 2010; Karimi et al., 2021; Minciuna et al., 2022). Hepatic fat acted as an energy reserve in most vertebrates during starvation (Browning et al., 2012; Mattson et al., 2018). Hepatic KLF15 expression was regulated by glucagon and insulin, which was induced during fasting or inhibited during feeding (Yamamoto et al., 2004; Teshigawara et al., 2005; Li et al., 2020). High-throughput proteomic analysis of liver samples obtained from adult giant salamanders during fasting for 3, 7, and 11 months revealed fasting activated not only the fatty acid oxidation and ketogenesis-related transcription factors PPAR-α and PPARγ-C1α, but also the gluconeogenesis-related transcription factors forkhead box O-class 1 (FoxO1), hepatocyte nuclear factor 4 alpha (HNF4α), and KLF15 (Geng et al., 2020). In the process, glucose was supplied to the blood using free amino acids produced by proteolysis in muscle and other tissues through glycogenolysis and gluconeogenesis in the liver (Oh et al., 2013; Melkonian et al., 2022). The FoxO1/3a-KLF15 pathway was activated by attenuating insulin signaling. FoxO1/3a transcriptionally regulated KLF15 level through directly binding to the liver-specific KLF15-1a promoter to promoted gluconeogenesis from amino acids (Takeuchi et al., 2021). It was also shown that fasting with/without forskolin treatment notably increased the expression of hepatic fibroblast growth factor 21 (FGF21) by enhancing the b-cell translocation gene 2 (BTG2)-KLF15 signaling network (Kim et al., 2019). KLF15 also induced Phosphoenolpyruvate carboxykinase (PEPCK) gene transcription in hepatocytes (Teshigawara et al., 2005). In addition, sterol-regulatory element binding proteins-1 (SREBP-1) was a key regulator of adipogenesis and prevented the conversion of glucose to triglycerides. Recent studies have shown that KLF15 inhibited SREBP-1 transcription by interaction with the LXR/RXR/RIP140 complex. These complexes recruited co-repressor RIP140 instead of co-activator steroid receptor coactivator-1 (SRC1), leading to a reduction in SREBP-1and downstream lipogenic enzyme level during early fasting or positive glycemic periods prior to hypoglycemia and PKA activation (Takeuchi et al., 2016). Furthermore, Foxo1/3a increased amino acid catabolic enzymes and decreased SREBP-1 levels through regulating KLF15, leading to accelerated amino acid breakdown and inhibition of adipogenesis during fasting (Takeuchi et al., 2021). ALL in all, the hepatic KLF15 is beneficial for the integration and regulation of metabolism among three macronutrients: protein, carbohydrate, and fat during fasting (Figure 3).

### KLF15regulates gluconeogenesis and amino acid catabolism

The liver plays an important role in fuel metabolism (Han et al., 2016). The liver was not only glycogen reservoir from which free glucose can be released to the circulation, but also the main site for gluconeogenesis (Radziuk and Pye, 2001; Oh et al., 2013; Zhang et al., 2018). Mice lacking factors (such as PEPCK) that regulated enzymes activity in the gluconeogenic pathway developed fasting hypoglycemia (Semakova et al., 2017). Recent studies have shown that mice with KLF15-targeted deletion (KLF15^−/−^) caused severe hypoglycemia after overnight (18 h) fasting compared with control animals. Evidence suggested that defects in amino acid catabolism, leading to fasting hypoglycemia at KLF15^−/−^ mice through limiting the availability of gluconogenic substrates. In KLF15^−/−^ mice, enhanced glucose clearance was primarily due to decreased hepatic glucose production. KLF15^−/−^ mice showed decreased expression of enzyme genes, which mediated amino acid degradation, such as alanine aminotransferase 1 (ALT1), 4-hydroxy-phenylpyruvate dioxygenase (HPD), proline dehydroge-nase (ProDH), and tryptophan 2,3-dioxygenase (TDO2). In addition, ornithine acylaminotransferase (OTC) mRNA level, one of the six enzymes in the urea cycle, was significantly reduced in KLF15^−/−^ mice. Finally, the enzymatic activity of ALT, which converted the key gluconeogenic amino acid alanine to pyruvate was reduced (−50%) at KLF15^−/−^ liver cells. Consistent with this observation, intraperitoneal administration of pyruvate, but not alanine, rescued fasting hypoglycemia at KLF15^−/−^ mice (Gray et al., 2007).

Acute depletion of KLF15 by RNAi inhibited gluconeogenic or amino acid-degrading enzyme levels, such as PEPCK or Glucose 6 phosphatase (G6Pase) in cultured hepatocytes (Wang and Dong, 2019). KLF15 binds to PEPCK gene promoter region and collaboratively regulated PEPCK gene level with transcriptional coactivator peroxisome proliferator-activated receptor-gamma co-activator 1-alpha (PGC1α). In addition, acute ablation of KLF15 specifically in the liver resulted in inhibition of gluconeogenic gene level. This result suggests that KLF15 is involved in the regulation gluconeogenesis gene. However, metformin accelerated the degradation of KLF15 expression, possibly by the promotion its ubiquitination (Takashima et al., 2010).

Fasting or forskolin (FSK) treatment of elevated Cereblon (CRBN) expression can enhanced the expression and secretion of hepcidin gene induced by liver gluconeogenesis signal by promoting the expression of KLF15. In addition, inhibition of CRBN and KLF15 significantly repressed hepcidin gene level, ultimately decline hepcidin secretion during FSK treatment. These results show that CRBN and KLF15 are mediators of fasting-induced hepatic hepcidin level and biosynthesis (Jo et al., 2021). Moreover, In KLF15 knockout mice cystathionine gamma-lyase was transcriptionally regulated by KLF15, while aspartic aminotransferase was regulated independently of KLF15 pathway (Mehrazad Saber et al., 2021).

### KLF15 and lipid

Previous studies reported that KLF15 was involved in regulating adipogenesis (Fan et al., 2022; Hu et al., 2022; Raza et al., 2022). In high-fat feeding, KLF15^−/−^ mice were resistant to hepatic insulin resistance and fatty liver and responded to pharmacological induction of endoplasmic reticulum stress. Deletion of the KLF15 gene can improve insulin resistance in mice under the influence of high-fat diet (HFD) but does not affect endoplasmic reticulum stress and hepatic inflammatory response with insulin resistance. Therefore, gene intervention targeting the KLF15 gene can improve HFD-induced insulin resistance. In addition, after endoplasmic reticulum stress was activated, the liver stress response of KLF15^−/−^ mice was significantly reduced when hepatic steatosis and insulin resistance were induced. However, inhibition of the KLF15 gene promotes C-Jun N-terminal kinase phosphorylation expression (Lee et al., 2016). Thus, deletion of KLF15 gene may lead to insulin resistance or steatosis at obese patients with HFD and uncoupling of endoplasmic reticulum stress and inflammatory response. In KLF15^−/−^ mice, enhanced fatty acid oxidation inhibited the mTORC1 signaling pathway, thereby improving hepatic steatosis. Therefore, the KLF15 gene is a key factor in regulating liver metabolism, and interference with the expression of the KLF15 gene may result in changes in HFD-induced liver lesions (Jung et al., 2013). Hepatocyte KLF15 controlled plasma corticosteroid transport through direct or specific transcriptional activation of Serpina6 encoding corticosteroid-binding globulin, thereby regulating inflammatory homeostasis (Jiang et al., 2022).

### KLF15 and bile acids (BAs)

BAs play an important role not only in promoting the intestinal absorption and transport of lipids and nutrients, but also in acting as a complex molecular signaling system (Thomas et al., 2008; McGlone and Bloom, 2019; Collins et al., 2022). Increasing evidence suggested that BAs levels were closely related to metabolic diseases, including fatty liver diseases, diabetes and arteriosclerosis (Chiang, 2009; Trauner et al., 2010). Recent studies showed that KLF15 was a necessary regulator of expression of key BAs synthase, BAs pool and fat absorption (Han et al., 2015; Wang et al., 2020). KLF15 deficiency disrupted circadian level in essential BAs synthetic enzymes, tissue BAs level and triglyceride/cholesterol absorption by negatively regulating FGF15 (Han et al., 2015). Similarly, Wang et al. reported Dipeptidyl peptidase-4 inhibitor teneligliptin improved obesity and other metabolic disorders. Mechanistically, teneligliptin increased the BAs synthesis by regulating CYP7A1 and CYP7B1 expression, which was mediated by the PI3K/AKT/KLF15/FGF15 signaling pathway. (Wang et al., 2020). Together, KLF15 may be a major therapeutic target for BAs-related metabolic diseases.

### KLF15 and endobiotic and xenobiotic metabolism (EXM)

The hepatic EXM machinery controls biotransformation and elimination of numerous endogenous (steroid sand bile acids) and exogenous (drugs and toxins) substances, thus ensuring homoeostasis and health (Plant and Klaassen, 2008; Woolbright and Jaeschke, 2015). The EXM process was divided into three phases, namely, substrate modification (phase I), conjugation (phase II), and excretion (phase III), which converted lipophilic compounds to hydrophilic products that were released to the circulatory system and cleared in urine and/or feces (Vasiliou et al., 2004; Ross and Crow, 2007; Han et al., 2019). The transcription factor KLF15 controlled all three stages of the EXM machinery through direct and indirect pathways, supported by unbiased transcriptomic analyses and confirmatory studies of cells, human tissues, and animals (Han et al., 2019). KLF15 inhibited phase I–II targets (Sult1a1and Cyp2b10), but induced Cyp2e1. Previous studies have shown that pregnane X receptor (PXR), constitutive androstane receptor (CAR), pregnane X receptor (FXR) and nuclear factor E2-related factor 2 (NRF2) were key transcriptional regulators of EXM of humans and rodents (Prakash et al., 2015). KLF15 deficiency moderately induced PXR and NRF2 level, but inhibited CAR level and has no effect on FXR. Consistent with elevated expression of PXR and NRF2, the binding of this factor to identified target genes Cyp3a11 and glutathione-s-transferase p1 (Gstp1) was increased at KLF15-deficient mouse liver. Liver-specific KLF15 deficiency (Li-KO KLF15^−/−^) mice altered the level of numerous phase I–III target genes and improved the pathologic progression of BAs and acetaminophen toxicity. In addition, Li-KO KLF15^−/−^) mice promoted degradation and elimination at endogenous steroid hormones, including testosterone and glucocorticoid, leading to decreased male fertility and blood glucose expression, severally. These phenotypes were reversed by viral recombination expressed in the liver KLF15 of Li-KO KLF15^−/−^ mice (Han et al., 2019).

**FIGURE 4:**
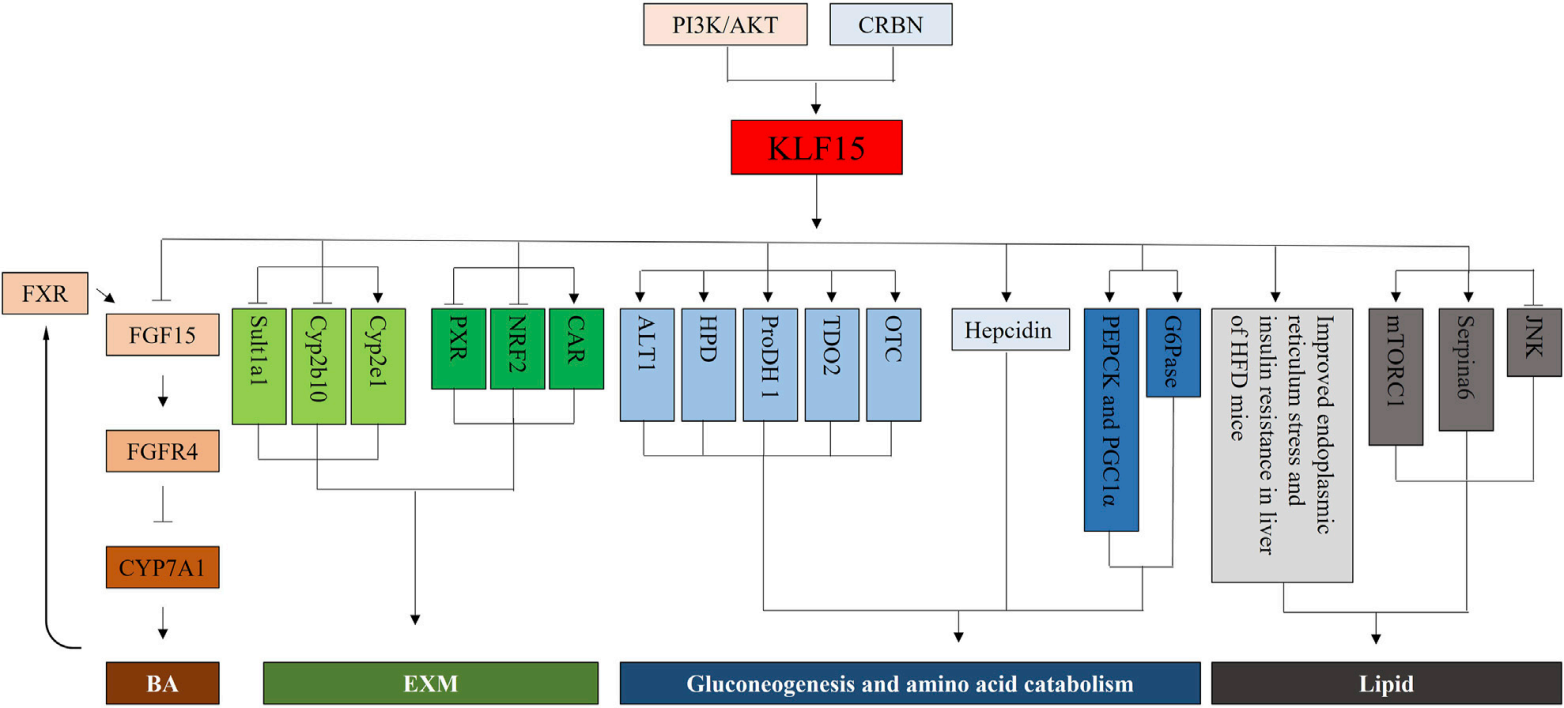
Mechanistic illustrations. First, KLF15, a direct negative regulator of ileal FGF15 and FGFR4, is critical for Cyp7a1 expression and BA synthesis in the liver. The BA synthesis is also mediated by PI3K/AKT/KLF15 signaling. Second, KLF15 represses phase I–II targets (Sult1a1, Cyp2b10) but induces Cyp2e1. And, KLF15 deficiency modestly enhances PXR and NRF2 expression, but decreases CAR. Third, KLF15^−/−^ mice manifests a decrease in the expression of genes for enzymes that mediate amino acid degradation, including those for ALT1, HPDProDH, TDO2, and OTC. Acute depletion of KLF15 by RNAi inhibits the expression of gluconeogenic or amino acid-degrading enzymes, such as PEPCK, PGC1α and G6Pase, in cultured hepatocytes. Moreover, CRBN and KLF15 are mediators of fasting-induced hepatic hepcidin expression and its biosynthesis. Finally, Deletion of the KLF15 gene improves endoplasmic reticulum stress and insulin resistance in liver of HFD mice. Inhibition of the KLF15 gene promotes the expression of JNK phosphorylation, and inhibits the mTORC1 signaling pathway. Hepatocyte KLF15 regulates plasma corticosteroid transport and thereby inflammatory homeostasis *via* direct and specific transcriptional activation of Serpina6.

In summary, mice with KLF15 deficiency were feasible, but unable to adapted to metabolic stress, including exercise or fasting, with phenotypes attributed into increased gene levels associated with gluconeogenesis, lipid metabolism, and branched-chain amino acid metabolism (Figure 4). Previous studies have suggested that KLF15 controlled nutrient acquisition (e.g., by control of bile acid production), inter-organ nutrient flux (e.g., branched-chain amino acid metabolism in muscle to assured optimal liver glucose production), and nutrient utilization (e.g., lipid utilization in muscle). These results demonstrate that KLF15 regulates glucose, lipid, and amino-acid metabolism, which in combination with current understanding of nutrient availability, suggesting that KLF15 is a vital node in the systemic metabolic homeostasis.

## The role and mechanism of KLF15 in liver diseases

### KLF15 and acute liver injury (ALI)

ALI, which was characterized by the sudden occurrence of massive hepatocyte necrosis or abnormal liver function, and eventually death due to complications such as multiple organ failure (Mooney, 2002; Kumar et al., 2019). In general, virus infection, drug toxicity, fatty liver damage, autoimmune disease and could lead to ALI (Vong et al., 2019). As a programmed cell death, apoptosis was thought to be an important mechanism in the development of ALI. Under normal conditions, cell apoptosis and proliferation maintain a dynamic balance, but under pathological conditions, this balance was broken, leading to hepatocyte apoptosis, which promoted liver injury (Guicciardi et al., 2013; Schwabe and Luedde, 2018). Hence, inhibition of apoptosis during ALI is the key to alleviating ALI. A recent study has reported that KLF15 was reduced in lipopolysaccharide (LPS)/D-galactosamine (D-GaIN) induced mice. Overexpression of KLF15 significantly inhibited the apoptosis and inflammation levels of liver *via* p38MAPK/ERK1/2 pathway (Tian et al., 2020). Another study also showed that knockdown of KLF15 promoted Bax expression, but BCL-2 level was inhibited by activating p53 pathway in LPS induced AML-12 cells (Tu et al., 2021). These results indicate that KLF15 may improve ALI by regulating apoptosis.

### KLF15 and hepatitis B virus (HBV)

HBV was an enveloped hepatotropic virus that caused cirrhosis and hepatocellular carcinoma (Tsai et al., 2018; Yu et al., 2022). However, available treatment options remain limited. It has been shown that food deprivation promoted HBV genes levels, which were reversible for refeeding. Moreover, part of the HBV activation may be related to fasting-induced KLF15 activation (Shlomai et al., 2006). Subsequent yeast one-hybrid screening assay further demonstrated that KLF15 induced the expression of HBV surface antigen (HBsAg) and the core protein and enhanced viral replication. Conversely, knockdown of KLF15 reduced viral gene expression and replication (Zhou et al., 2011). Therefore, inhibition of KLF15 may be a potential strategy for the treatment of HBV.

### KLF15 and autoimmune hepatitis (AIH)

**FIGURE 5:**
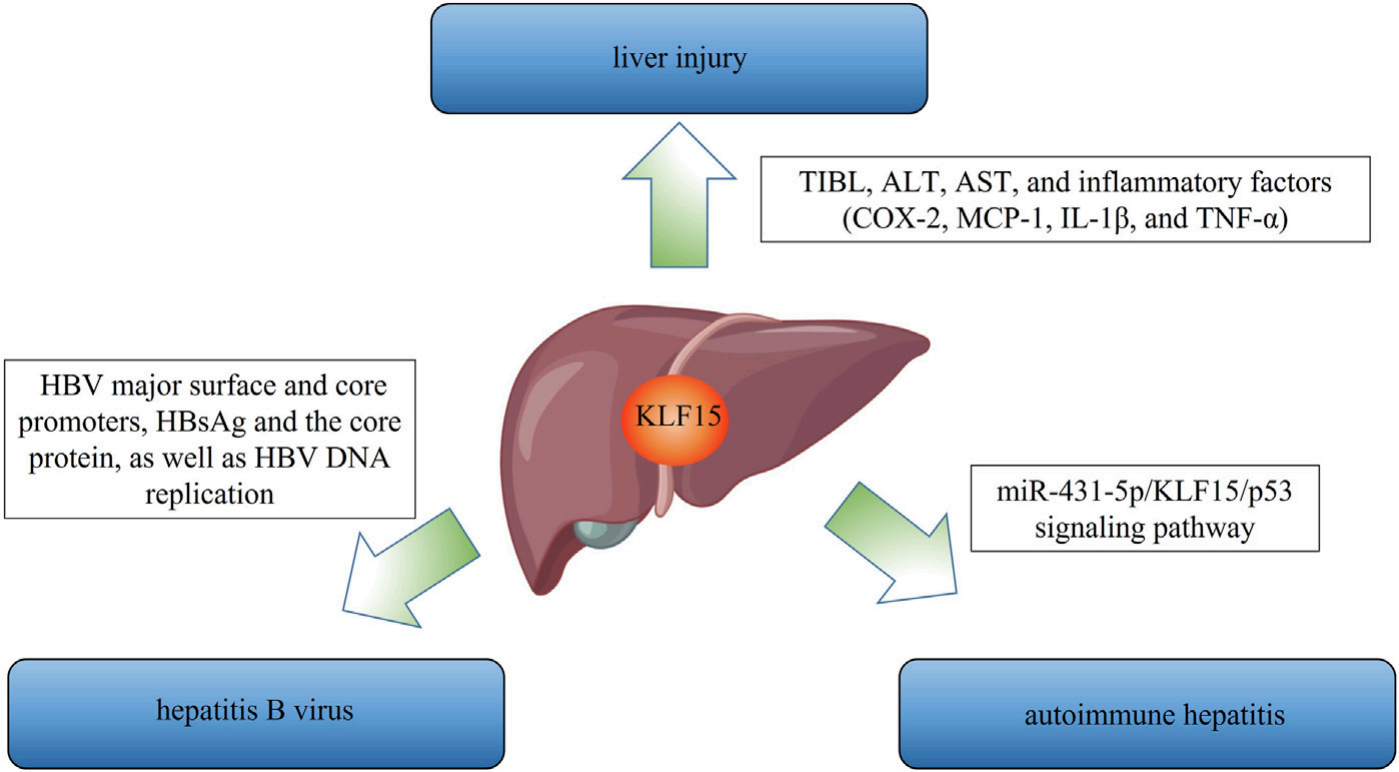
The multiple roles of KLF15 in liver disease are outlined. First, KLF15 plays an important role in acute liver injury, including the regulation of TIBL, ALT, AST, and inflammatory factors (COX-2, MCP-1, IL-1β, and TNF-α). With respect to hepatitis B virus, KLF15 positively regulates HBV major surface and core promoters, HBsAg and the core protein, as well as HBV DNA replication. Finally, the miR-431-5p/KLF15/p53 signaling pathway is a potential therapeutic target in autoimmune hepatitis.

AIH, a progressive inflammatory liver disease caused by inadequate autoimmune tolerance (Komori, 2021; Volk and Reau, 2021; Tan et al., 2022). The standard therapy for AIH was glucocorticoid alone or in combination with azathioprine. However, more than 20% of patients do not respond well and end-stage patients still require liver transplantation. A recent study showed that downregulation of miR-431-5p inhibited apoptosis *via* KLF15/p53 pathway in S100 induced AIH mice and KLF15 inhibition abolished this event. (Tu et al., 2021) (Figure 5). Although few studies focus on the role of KLF in AIH, current evidence tentatively suggests that KLF15 is a promising target in treating AIH.

## Conclusion and future perspective

KLF15 is a basic metabolic regulator of all major nutrient classes and tissues. In this review, we summarized the role and mechanism of KLF15 in metabolic reprogramming, which provided new insights for the progression and treatment of liver diseases. Moving forward, the upstream media of KLF15 should be elucidated. In other words, how the physiological state and metabolic environment of the body control KLF15 expression and function? Furthermore, whether KLF15 is involved in the treatment of liver diseases with other metabolic transcriptional regulators? KLF15 is expected to be a potential therapeutic target for hepatic metabolism disorder. To verify the particular functional mechanism of KLF15 in different cell types or diseases will be a promising research direction for advancing precision medicine.

## Glossary

WT1: Wilms Tumor 1
CtBP: C-terminal binding protein
Tat: tyrosine aminotransferase
Cyp2b10: cytochrome P450 2b10
FGF21: fibroblast growth factor 21
SRC1: steroid receptor coactivator-1
AIH: autoimmune hepatitis
ALI: acute liver injury
ALT1: alanine aminotransferase 1
BAs: bile acids
BCAA: branched-chain amino acid
CRBN: Cereblon
CREB: cAMP response element binding protein
ECM: extracellular matrix
EXM: endobiotic and xenobiotic metabolism
FoxO1: forkhead box O-class 1
FSK: forskolin
G6Pase: Glucose 6 phosphatase
GLUT4: glucose transporter 4
HBV: hepatitis B virus
HFD: high-fat diet
HPD: 4-hydroxy-phenylpyruvate dioxygenase
HNF4α: hepatocyte nuclear factor 4 alpha
iPSCs: induced pluripotent stem cells
KLFs: Krüppel-like factors
KLF15: Krüppel-like factor 15
Li-KO: Liver-specific KLF15 deficiency
OSM: Oncostatin M
OTC: ornithine acylaminotransferase
PEPCK: Phosphoenolpyruvate carboxykinase
ProDH: proline dehydroge-nase
PPARγ: peroxisome proliferator-activated receptor gamma
Sin3A: SIN3 transcription regulator family member A
BTG2: b-cell translocation gene 2
PGC1α: peroxisome proliferator-activated receptor-gamma co-activator 1-alpha
SREBP-1: sterol-regulatory element binding proteins- 1
TDO2: tryptophan 2,3-dioxygenase
PXR: pregnane X receptor
CAR: constitutive androstane receptor
FXR: pregnane X receptor
NRF2: nuclear factor E2-related factor 2
Gstp1: Glutathione-S-transferase P1
LPS: lipopolysaccharide
D-GaIN: D-galactosamine
